# Bridging intestinal immunity and gut microbiota by metabolites

**DOI:** 10.1007/s00018-019-03190-6

**Published:** 2019-06-27

**Authors:** Gang Wang, Shuo Huang, Yuming Wang, Shuang Cai, Haitao Yu, Hongbing Liu, Xiangfang Zeng, Guolong Zhang, Shiyan Qiao

**Affiliations:** 1 State Key Laboratory of Animal Nutrition, College of Animal Science and Technology, China Agricultural University, Beijing, 100193 China; 2 Beijing Key Laboratory of Biological Feed Additive, China Agricultural University, Beijing, 100193 China; 3 Department of Animal Science, Oklahoma State University, Stillwater, OK 74074 USA

**Keywords:** Fecal microbiota transplantation, Innate and adaptive immune cells, Germ-free animals

## Abstract

The gastrointestinal tract is the site of nutrient digestion and absorption and is also colonized by diverse, highly mutualistic microbes. The intestinal microbiota has diverse effects on the development and function of the gut-specific immune system, and provides some protection from infectious pathogens. However, interactions between intestinal immunity and microorganisms are very complex, and recent studies have revealed that this intimate crosstalk may depend on the production and sensing abilities of multiple bioactive small molecule metabolites originating from direct produced by the gut microbiota or by the metabolism of dietary components. Here, we review the interplay between the host immune system and the microbiota, how commensal bacteria regulate the production of metabolites, and how these microbiota-derived products influence the function of several major innate and adaptive immune cells involved in modulating host immune homeostasis.

## Introduction

Commensal bacteria, with an estimated > 10^13^ cells in the mammalian gastrointestinal tract, are key players in the maintenance of stable gut physiology and organismal homeostasis. The gut microbiota is not only essential for the digestion, absorption, and storage of food substrates, but also for host immune system development, especially with regard to regulating the homeostasis of the gut immune system [1]. Moreover, balanced immune homeostasis may promote and limit the growth of beneficial and harmful microbes, respectively [2].

The mammalian immune system has coevolved with the complex community of indigenous microbiota that constitutively colonize all barrier surfaces [3]. Indeed, as opposed to existing in isolation, microorganisms regulate multiple aspects of host functions, including metabolism and immune system development [4]. The interaction between the microbiota and host is most prominent on the mucosal surface, which provides an interface between the largely microbe-free host, the microbiota, and the environment. Multiple mucosal defenses maintain compartmentalization of the gut microbiota in the intestinal lumen, such as a specialized mucosal layer, antimicrobial peptides, secreted immunoglobulins and a diverse array of mucosal lymphocytes [5]. Specific changes in gut microbiota composition may activate the mucosal immune system, resulting in chronic inflammation and the development of mucosal injury [6].

Overall, modulation of host metabolism and immunity by gut microorganisms depends mainly on the exchange of small molecules, termed metabolites, between the gut lumen and the epithelial surface. The gut microbiota synthesizes, modulates, and degrades a variety of metabolites, thereby acting as a functional complement to host metabolism, especially for dietary components that the host cannot metabolize [7]. Most metabolites originate in one of two ways: (1) diet-dependent microbial products or (2) diet-independent microbial products. Diet-dependent microbial products are directly linked to diet or digestion; examples of such products include but are not limited to short-chain fatty acids (SCFAs), secondary bile acids, indole and indole derivatives. Diet-independent microbial products are synthesized de novo by gut microbes; examples of these products include lipopolysaccharides and peptidoglycans [8]. More than 50% of the metabolites in feces and urine are derived from or modified by the gut microbiota [9].

Recent studies have identified a critical role for the metabolites derived from commensal bacteria in modulating the homeostasis and function of innate and adaptive immune cells through indirect and direct mechanisms [10–12]. Small molecules diffuse directly through the hard mucosal layer to trigger epithelial signals via metabolite-specific receptors, thereby activating signal transduction pathways and transcriptional programs that control the differentiation, proliferation, maturation and effector functions of many cells. These sensing receptors are expressed in different combinations in mucosal cell subsets, such as intestinal epithelial cells (IECs), macrophages (MΦ), dendritic cells (DCs), T cells, and innate lymphoid cells (ILCs), and thus have a critical role in host-microbiota mutualistic interactions [13]. In addition, some metabolites may serve as signaling molecules for quorum sensing and interbacterial communication.

Although it is known that the gut microbiota modulates host biology in numerous ways, many known metabolites have not yet been functionally characterized, and the molecular mechanism involved in these reciprocal interactions is still poorly understood. Here, we summarize the current knowledge of the interaction between the gut microbiota and intestinal immunity, describe the production of several immunomodulatory metabolites, and highlight how these metabolites affect and regulate immune cells to maintain gut health.

## The gut microbiota and immune system development

The immune system is responsible for detecting and repairing physiological disorders through both immune responses and critical regulatory processes [14, 15]. However, relative to other organs, the intestinal immune system faces unique challenges resulting from continuous exposure to significant microbial loads. The gut microbiota does not simply evade the immune system to persist in the gastrointestinal tract, and recent research has shown that these microorganisms contribute to the development, maturation, and regulation of the immune system [16, 17]. Due to the abundance and diversity of the gut microbiota, it is important to identify individual species within these communities and their associated effects on the immune system, especially for uncultured or low-abundance microorganisms. Germ-free animals and fecal microbiota transplantation (FMT) are techniques that have been widely used to study the contribution of microbiota (individual species or healthy communities) to the host immune system.

### The immune system under a germ-free state

Germ-free animals have a severely immunodeficient immune system and thus, a higher susceptibility to infection, and this immunodeficiency is especially pronounced in the gut. For instance, germ-free animals exhibit low immunoglobulin A (IgA) concentrations in the intestinal lumen and underdeveloped of gut-associated lymphoid tissues, including small and few Peyer’s patches (PP) and mesenteric lymph nodes (MLNs) [16, 18, 19]. In addition, convincing evidence derived from studies on germ-free animals suggests that commensal intestinal bacteria are essential for the development and function of lymphocyte subsets. *Bifidobacterium adolescenti*s and segmented filamentous bacteria (SFBs) have been identified as potent inducers of IL-17-producing T helper (Th) 17 cells [20, 21]. Mice lacking SFBs among gut microbiota have fewer Th17 cells than mice with abundant SFBs, and normal development of Th17 cells can be restored by SFB supplementation [22]. Similarly, monoassociations of germ-free mice with specific *Clostridia* and *Bacteroides* species can promote the differentiation of regulatory T cells (Tregs) and repress Treg reduction in the lamina propria (LP) of germ-free mice [23, 24]. Intestinal microbial species were found to be targeted and coated by secretory IgA and alteration of the IgA response leads to reductions in overall microbial diversity. Secretory IgA in the gut enhances the translocation of microbes into lymphoid tissues to facilitate antigen presentation [25–27]. The development and maturation of IgA-producing plasma cells are deficient in the gastrointestinal tract of germ-free mice, and IgA levels are thus low [28–30]. The intestinal mucus layer, which acts as a barrier to protect the epithelium from irritation by the luminal contents, is thinner in germ-free animals than in conventional animals [31, 32], but its thickness can be recovered after colonization with *Lactobacillus plantarum* [33].

### Immune system alteration during dysbiosis and balance

Antibiotics are one of the greatest achievements in the history of medicine, but their long-term application leads to disruption of intestinal microbial communities [34]. Moreover, many antibiotic compounds increase the host’s susceptibility to several pathogens such as *Salmonella* species and vancomycin-resistant *Enterococcus* spp. [35].

In general, profound evidence demonstrates the impact of antibiotic treatment on the intestinal ecosystem and immune system. Mice treated with vancomycin or colistin from birth display a reduced number of isolated lymphoid follicles in the small and large intestine [36]. In addition, mice treated with a mixture of antibiotics consisting of vancomycin, neomycin, and metronidazole exhibit reduced antimicrobial peptide expression [37]. After microbiota depletion by broad-spectrum antibiotic treatment, the Treg cell populations in MLNs, PPs and the colon LP are also reduced, although recolonization by conventional microbiota strains or select species of bacteria can restore these cell populations [38, 39]. Similarly, compared with nontreated mice, antibiotic-treated mice have fewer mucosal Th17 cells and ILCs, which play roles in resistance to extracellular microbiota and pathogenesis by secreting IL-17 and IL-22 [38, 40]. Overall, the impacts of antimicrobial treatment on the immune system are mainly due to changes in microbial community composition.

FMT refers to the entire transfer of fecal microbiota from a healthy donor to a recipient’s intestinal tract to normalize the composition and functionality of the intestinal microbiota. FMT can be applied to normalize the composition of the gut microbiota and increase the proportion and diversity of beneficial bacteria, thereby reducing gut inflammation. FMT also provides the signals necessary for epithelial regeneration, induces the production of mucins and antimicrobial peptides, and reduces bowel permeability to maintain epithelial barrier integrity [41, 42]. Furthermore, FMT stimulates the intestinal adaptive immune response through the Toll-like receptor (TLR) pathway to promote the synthesis of immunoglobulins (e.g., IgA, IgG, and IgM), thereby protecting the intestinal mucosa [43].

Ira Ekmekciu et al., showed treating conventional C57BL/6j mice with a broad-spectrum antibiotic for 8 weeks profoundly changed the immune cell repertoire, and the CD4^+^ cell production of some cytokines (IFN-γ, IL-17, IL-22 and IL-10) declined. FMT can reconstitute the intestinal microbiota and restore the numbers of CD4^+^, CD8^+^, and B220^+^ cells in the small intestine and colonic CD4^+^ cells as early as 7 days after transfer [44]. The same author conducted a similar study to investigate the abilities of the FMT of *Escherichia coli*, *Lactobacillus johnson* and complex murine microbiota to restore immune function in mice that were immunosuppressed by antibiotic-induced microbiota depletion. Compared with the administration of individual commensal bacteria, FMT appears to be more effective at restoring the decreased numbers of cytokine-producing CD4^+^ T cells in the mucosal and systemic compartments and maintaining immune functions [45]. Similar experiments in a piglet model also indicated that FMT can modulate the metabolic function of the gut microbiota and enhance the microbiota-derived catabolite tryptophan, increasing the production of IL-22 to maintain the intestinal barrier of newborn piglets [41].

## Production of microbiota-derived metabolites

Typically, the relationship between the host and the gut microbiota is highly mutualistic. In general, the host provides food and shelter for bacteria, which in turn helps the host digest complex foods and synthesize essential nutrients (e.g., vitamins B and K) [46]. The host may in fact rely on the gut microbiota for digestion and metabolism. Through the fermentation of undigested dietary components that reach the large intestine, as well as endogenous compounds generated by the microbial communities, the gut microbiota produces an extraordinarily wide range of metabolites [4].

As mentioned above, most metabolites originate from diet-dependent and diet-independent sources [8]. The latter metabolites (e.g., ATP and polysaccharide A) are synthesized de novo by gut microbes. Diet-dependent microbial products can be broadly classified into two categories: (1) directly generated digestion or fermentation of dietary components by the gut microbiota and (2) products of host metabolism, that are biochemically modified by the gut microbiota. Using mass spectrometry, Matsumoto et al., found 179 metabolites in the colonic lumens of mice, but 48 were not present in the food consumed by these mice; among these metabolites, 35 had differential concentrations between the colons of germ-free mice and conventional mice [47]. In fact, nearly 10% of metabolites in the blood as well as more than 50% of those in the feces and urine are derived from or modified by the gut microbiota [9, 48]. In the following sections, we describe the production of the most prominent bacteria-derived diet-dependent metabolites.

### Microbial modification of dietary component derived metabolites

#### Short-chain fatty acids

SCFAs are volatile fatty acids with a 1–6 carbon atoms backbones and are produced in the large intestine through the bacterial fermentation of undigested polysaccharides. Members of Firmicutes are the main producers of butyrate, whereas Bacteroidetes produces most acetates and propionates [85, 86]. Several bacterial species have been reported to be capable of producing SCFAs (Table 1).

**Table 1:** The microbial metabolites catalyzed form gut microbiota

Substrate	Metabolite	Producers	References
Polysaccharides	Acetate	*Blautia hydrogenotrophica*, *Methanobrevibacter smithii*, *Ruminococcus gnavus*, *Eubacterium hallii*, *Eubacterium cylindroides*, *Faecalibacterium prausnitzii*	[49–51]
	Propionate	*Ruminococcus obeum*, *Coprococcus catus*, *Roseburia inulinivorans*, *Phascolarctobacterium succinatutens*, *Salmonella enterica*, *Eubacterium hallii*	[50, 51, 52]
	Butyrate	*Clostridium acetobutylicum*, *Butyrivibrio fibrisolvens*, *Faecalibacterium prausnitzii*, *Roseburia intestinalis*, *Ruminococcus gnavus*, *Eubacterium ruminantium*, *Eubacterium hallii*, *Eubacterium cylindroides*, *Faecalibacterium prausnitzii*, *Roseburia inulinivorans*, *Eubacterium rectale*, *Anaerostipes spp*., *Coprococcus comes*, *Coprococcus eutactus*, *Coprococcus catus*	[51–56]
Tryptophan	Indole	*Desulfitobacterium hafniense*, *Clostridium malenomenatum*, *Clostridium limosum*, *Clostridium lentoputrescens*, *Clostridium tetani*, *Bacteroides sp*., *Flavobacterium sp*.	[57–60]
	Indoleacetic acid (IAA)	*Bacteroides fragilis*, *Bacteroides thetaiotaomicron*, *Bifidobacterium longum subsp*. *Longum*, *Bifidobacterium pseudolongum*, *Clostridium difficile*, *Clostridium paraputrificum*, *Clostridium perfringens*, *Peptostreptococcus asaccharolyticus*, *Clostridium sticklandii*, *Clostridium putrefaciens*	[58, 61]
	Indoleacrylic acid (IA)	*Clostridium sporogenes*, *Peptostreptococcus russellii*, *Peptostreptococcus anaerobius*, *Peptostreptococcus stomatis*	[62, 63]
	Indolelactic acid (ILA)	*Bacteroides fragilis*, *Bacteroides thetaiotaomicron*, *Bifidobacterium bifidum*, *Bifidobacterium adolescentis*, *Bifidobacterium longum subsp. Infantis*, *Bifidobacterium longum subsp. Longum*, *Bifidobacterium pseudolongum*, *Clostridium perfringens*, *Peptostreptococcus asaccharolyticus*, *Clostridium sporogenes*, *Lactobacillus paracasei*, *Lactobacillus reuteri*, *Lactobacillus murinus*, *Bifidobacterium spp*	[61, 62, 64–66]
	Indolepropionic acid (IPA)	*Clostridium sporogenes*, *Clostridium botulinum*, *Clostridium caloritolerans*, *Peptostreptococcus russellii*, *Peptostreptococcus anaerobius*, *Peptostreptococcus stomatis*	[58, 63]
	Skatole	*Clostridium drakei*, *Clostridium scatologenes*, *Lactobacillus spp.*	[67, 68]
	Indolealdehyde (IAld)	*Lactobacillus reuteri*, *Lactobacillus murinus*	[65]
	Tryptamine	*Clostridium sporogenes*, *Ruminococcus gnavus*	[69]
Bile acids	Secondary bile acids	*Clostridium scindens*, *Ruminococcus gnavus*, *Bacteroides fragilis*, *Bacteroides vulgatus*, *Clostridium perfringens*, *Peptostreptococcus productus*, *Pseudomonas testosteroni*, *Lactobacillus plantarum*, *Bifidobacterium*, *Clostridium hiranonis*, *Eubacterium*	[70–77]
	Oxo- (or keto) bile acids	*Eubacterium lentum*, *Clostridium perfringens*, *Ruminococcus. ganvus*	[78–80]
Arginine	Polyamines	*Bacteroides fragilis*, *Shigella flexneri*, *Streptococcus pneumoniae*	[81–83]
Histidine	Histamine	*Lactobacillus rhamnosus*	[84]

Acetate is the most abundant SCFA in the colon, accounting for more than half of the total SCFAs detected in feces [87]. Production of acetate by gut bacteria involves two major metabolic pathways. The majority of acetate is the byproduct of undigested polysaccharide fermentation by most enteric bacteria. In addition, nearly one-third of acetate is derived from acetogenic bacteria, such as *Blautia hydrogenotrophica*, which can use H_2_ and CO_2_ or formic acid to synthesize acetate via the Wood-Ljungdahl pathway [49, 50].

Three main pathways participate in the formation of propionate by gut bacteria: the succinate pathway, the acrylate pathway and the propanediol pathway. Most propionate is formed by Bacteroidetes while utilizing the succinate pathway as a substrate. The acrylate pathway is used to convert lactate to propionate through several enzymatic reactions, and this pathway appears to be limited to a few members of the families Veillonellaceae (e.g., *Megasphaera* spp.) and Lachnospiraceae (e.g., *Coprococcus catus* and *Clostridium lactatifermentans*) [85]. The propanediol pathway is involved in the conversion of deoxy-sugars (rhamnose and fucose) to propionate and can be found in Proteobacterium *Salmonella enterica* serovar Typhimurium and *Lachnospiraceae* bacteria [88, 89].

The production of butyrate originates from two molecules of acetyl-CoA and two different pathways for the liberation of butyrate from butyryl-CoA: the acetate CoA-transferase pathway and the butyrate kinase pathway [51, 56]. The latter converts butyryl-CoA into butyrate using phosphotransbutyrylase and butyrate kinase. However, this pathway is not common and is mainly limited to some members of *Coprococcus* (e.g., *Coprococcus eutactus* and *Coprococcus comes*) [85]. The butyryl-CoA:acetate CoA-transferase route is utilized in most known butyrate-producing gut strains (e.g., *Eubacterium rectangle, Roseburia* spp. *Coprococcus cactus*, *Faecalibacterium prausnitzii, Anaerostipes* spp. and *Eubacterium hallii*) [89].

The production of SCFAs by bacteria is not unique. Depending on the growth substrate, the fermentation products produced by bacteria can change. For instance, butyrate can be found when *Roseburia inulinivorans* is cultured with glucose, whereas propionate, propanol and butyrate are produced when cultured with fructose [89]. Although carbohydrates are the main source of SCFAs, branched-chain amino acids, known as branched-chain SCFAs (BSCFAs), can be converted into isobutyrate, isovalerate, and 2-methyl butyrate, but they contribute very little (5%) to total SCFA production [90].

#### Tryptophan metabolites

**Fig. 1:**
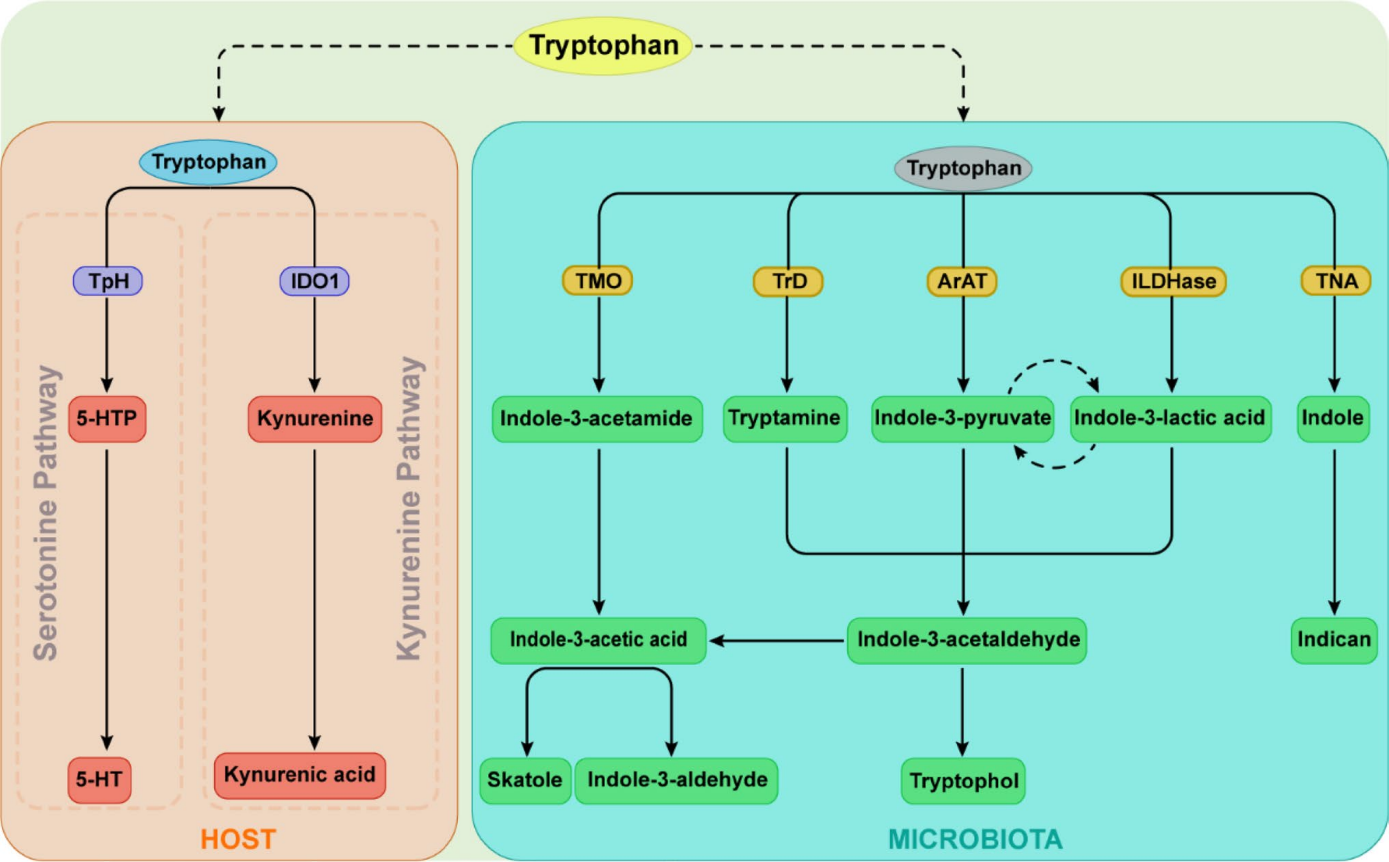
Tryptophan metabolic pathways in the host and microbiota. Among microbial metabolites, indole and indolic acid derivatives are the predominant Trp microbial metabolites in the gut, and the intestinal microbiota produce different metabolites based on which catalytic enzymes the bacteria produce. The kyn and serotonin pathways are the primary routes of host Trp metabolism. *Trp* tryptophan, *TpH* trytophan hydroxylase, *5-HTP* 5-hydroxy tryptophan, *5-HT* serotonin, *IDO1* indoleamine 2,3-dioxygenase, *TMO* tryptophan decarboxylase, *TrD* tryptophan decarboxylase, *ArAT* aromatic amino acid aminotransferases, *ILDHase* indole-3-lactic acid dehydrogenase, *TNA* tryptophanase

The gastrointestinal tract harbors billions of gut microorganisms that contribute to the first-pass metabolism of dietary components. Tryptophan is utilized to synthesize proteins, yet intestinal bacteria can directly utilize this amino acid to produce many immunologically important metabolites [65, 91], with indole, indolic acid derivatives and tryptamines being the main microbial tryptophan metabolites in the gut [92] (Fig. 1). In addition, indole and indolic acid derivatives can be further metabolized into other final products, such as the conversion of indole-3-acetate to skatole or indole-3-aldehyde, indole to indicant, and indole acrylic acid to indole propionic acid [93] (Fig. 1).

Many bacterial species can convert tryptophan into indole and indole derivatives (Table 1). As one of the major bacterial tryptophan metabolites, indole has been recognized as an important interspecies and interkingdom signaling molecule for biofilm formation as well as for regulating bacterial motility and resisting the invasion of nonindole-producing species, such as *Salmonella enterica* and *Pseudomonas aeruginosa johnsonii*) [94]. Through the activity of the enzyme tryptophanase, a variety of bacteria, including *Achromobacter liquefaciens*, *Paracolobactrum coliforms*, *Bacteroides* spp. and *E. coli*, can convert tryptophan into indole, and a variety of tryptophan microbial metabolites are indolic acid derivatives, such as indole-3-aldehyde, indole lactic acid, indole-3-acetic acid and indole acrylic acid [95]. Via aromatic amino acid aminotransferase, *Lactobacillus reuteri and Lactobacillus johnsonii* convert tryptophan to indole-3-aldehyde which contributes to the maintenance of intestinal homeostasis by preventing the colonization of pathogenic microorganisms (such as *Candida albicans*) and weakening inflammatory disorders affecting the intestinal tract [65]. Skatole is not directly synthesized from tryptophan but is decarboxylated by indole-3-acetic acid [92] (Fig. 1). Some species belonging to *Lactobacillus*, *Bacteroides* and *Clostridium* can convert indole-3-acetic acid into skatole [67, 96], which can affect the growth and reproduction of several bacteria, such as *Salmonella*, *Shigella* and *Escherichia* [92]. Tryptophan metabolism by gut bacteria involves multiple catalytic reactions; various bacteria may possess the same enzymes to produce the same metabolites, or a bacterium may produce different tryptophan metabolites. *Bacteroides* spp. and *E. coli* are capable of converting tryptophan to indole and tryptamine [95], whereas *Clostridia* can catabolize tryptophan to indole pyruvic acid, which is then converted to indole-3-acetic acid [93].

In addition to microbial-derived tryptophan metabolites, cells in the liver and extrahepatic tissues can generate tryptophan metabolites such as kynurenine (kyn) and serotonin (5-HT) [97] (Fig. 1). The kyn pathway is the primary route by which the host metabolizes tryptophan through indoleamine 2,3-dioxygenase (IDO1) in the intestine. IDO1 carries out the catabolic conversion of tryptophan to kyn, and kyn can then be used to generate kynurenic acid (KA) or xanthurenic acid (XA) via different routes [98]. Approximately 95% of the tryptophan ingested is broken down via the kyn pathway, ~ 1–2% is used for protein synthesis, and ~ 1–2% is used for the 5-HT pathway [99].

Unlike the relatively simple background of host endogenous tryptophan metabolism, the intestinal environment is extremely complex with regard to bacterial tryptophan metabolism, and many strains that possess catalytic enzymes capable of metabolizing tryptophan are still unknown. Therefore, substantially more research on tryptophan metabolizing bacteria is needed.

### Microbial modification of host-derived metabolites

#### Secondary bile acids

**Fig. 2:**
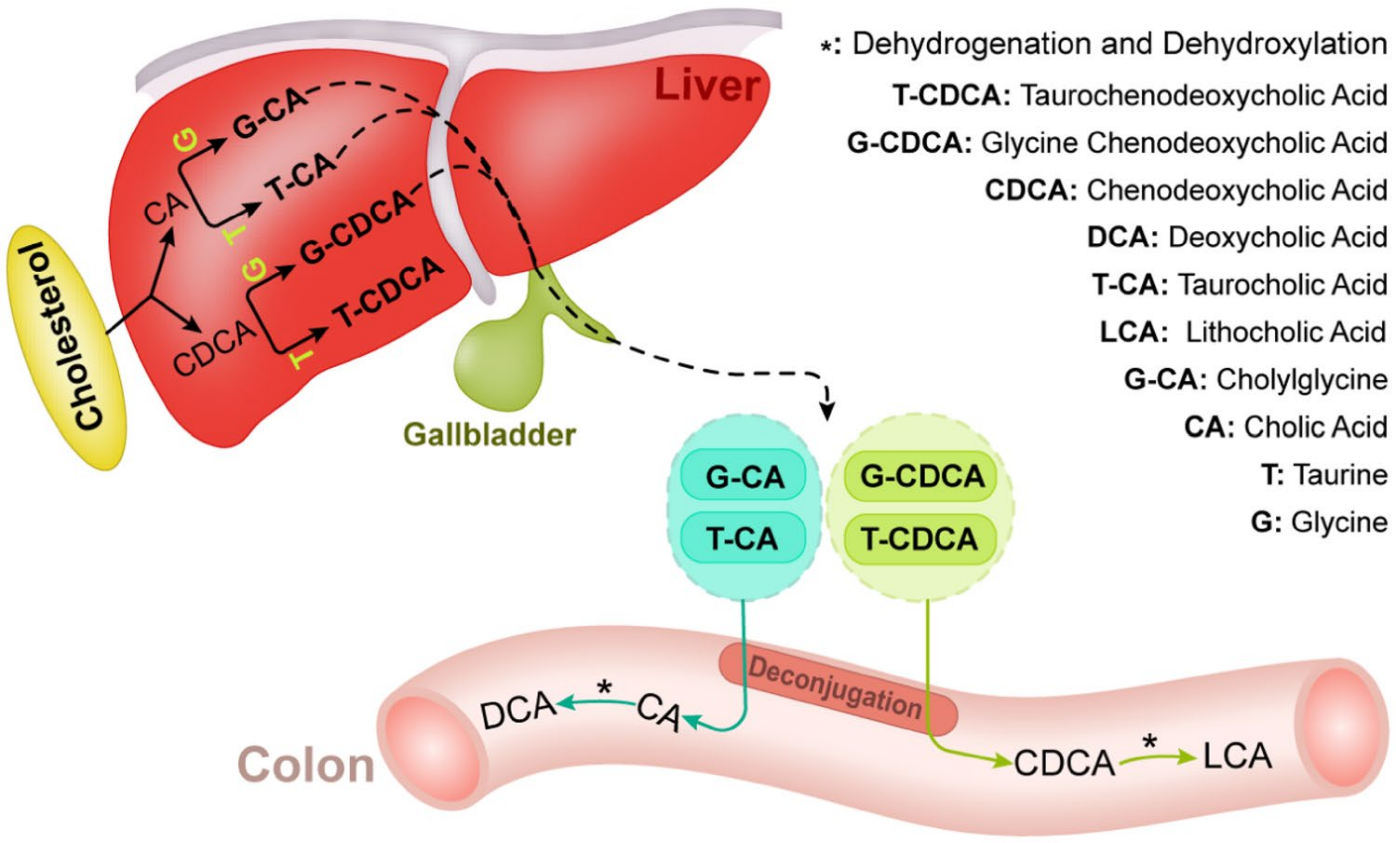
Biosynthesis of bile acids and microbial modification of bile acid metabolism. Cholesterol is converted into two primary bile acids, cholic acid (CA) and chenodeoxycholic acid (CDCA), in the liver and further conjugated to glycine or taurine. The bile salts that escape active transport in the distal ileum become substrates for biotransformation reactions by intestinal bacteria. In the intestine, especially in the colon, these conjugated species are first deconjugated to CA or CDCA, and then dehydrogenated and dehydroxylated by gut bacteria into secondary bile acids such as DCA and LCA

Bacterial metabolites are sourced from not only dietary components but also from substrates secreted into the gut lumen via host metabolism[100]. Primary bile acids, such as cholic acid (CA; 3α, 7α, 12α-trihydroxy-5β-cholan-24-oic acid) and chenodeoxycholic acid (CDCA; 3α, 7α-dihydroxy-5β-cholan-24-oic acid), are derived from cholesterol catabolism in the liver and are further secreted by the gall bladder into the intestine after conjugation to glycine (in humans) or taurine (in mice) [17] (Fig. 2). Nearly 95% of bile acids can be reabsorbed in their conjugated form in the terminal ileum through active transport via apical sodium-dependent bile acid transporters (ASBT) [101], and bile acids that escape active transport in the distal ileum become substrates of colonic bacteria for biotransformation reactions. Several bacteria that metabolize bile acids are shown in Table 1. Biotransformation of bile salts involves multiple steps and typically begins with the deconjugation of taurine or glycine by bile salt hydrolase (BSH) [102]. These deconjugated bile acids are further metabolized via 7-dehydroxylation into secondary bile acids by the microbiota. Lithocholic acid (LCA) and deoxycholic acid (DCA) are the main secondary bile acids resulting from unabsorbed chenodeoxycholic acid (CDCA) and CA, respectively [102–104]. The complicated 7-dehydroxylation process involves several reactions carried out by microbiota strains harboring bile acid-inducible (bai) genes [105]. Bacteria capable of producing secondary bile acids belong to the genera *Bacteroides*, *Lactobacillus*, *Bifidobacterium, Clostridium* (clusters XIVa and XI) and *Eubacterium* [76, 77, 102].

Oxo- (or keto) bile acids are other products of bile acid microbial biotransformation formed via gut microbial oxidation by hydroxysteroid dehydrogenases (HSDHs), which have been identified in Proteobacteria, Actinobacteria, and Firmicutes [106, 107]. As these oxidation reactions are reversible and epimerization may occur during the process, some iso-bile acids are found in the serum and urine, particularly in feces and colon digesta [108]. Bacteria capable of producing iso-bile acids include *Eubacterium lentum*, *Clostridium perfringens* and *Ruminococcus ganvus* [78–80]. Overall, microbial bile acid metabolism plays an important role in modulating the extent of bile acid recycling by the host.

### Metabolic cross-feeding

The intestine provides a nutrient-rich environment for its commensal microorganisms, which compete intensely with each other for nutrition and space but also cooperate in metabolism [109, 110]. Indeed, the metabolite signals derived from microbes function in not only the host but also in other microbiota strains, and the interactions between such communities may significantly effect microbial ecology and physiology. Bacteria can exchange metabolites through the extracellular environment and directly exchange nutrients through cell–cell connections [111]. Microbiota strains are found in a variety of communities in the gastrointestinal tract, and such communities are thought to be largely shaped by interspecies competition for available nutrients or syntrophic interactions (such as beneficial metabolic exchange); the latter can also be called metabolic cross-feeding [112]. Bacterial cross-feeding refers to the utilization of products from the metabolism of one bacteria by another.

Types of bacterial cross-feeding include metabolic cross-feeding and substrate cross-feeding; the former denotes the utilization of metabolic end products produced by a microbiota strain, whereas the latter denotes the utilization of intermediate products formed by a microorganism [113, 114]. Lactate, a common end product of bacterial fermentation produced by *Lactobacillus* and *Bifidobacterium*, can be used by other bacteria via metabolite cross-feeding to produce SCFAs. For instance, *E. hallii* cannot grow in pure culture in the presence of starch, but *B. adolescentis* grows well in the presence of lactate. When these two strains are co-cultured, the concentration of lactate significantly decreases, while that of butyrate increases. Thus, *E. hallii* can produce butyric acid using the lactose produced by *B. adolescentis*. A similar pathway can be found in the acetate producer *Desulfovibrio piger* and some Firmicutes bacteria [115, 116].

Substrate cross-feeding is more similar to the cooperation between multiple bacteria, such as *Roseburia* spp., which cannot utilize lactate for growth, but grows well by utilizing breakdown products, namely, fructose by *Bifidobacterium* species [113, 117]. *B. thetaiotaomicron* can utilize mucin glycoproteins to liberate free sialic acid, but it cannot further utilize sialic acid. In contrast, *C. difficile* is able to use sialic acid as a nutrient for growth, mainly relying on the sialic acid metabolic pathway [118]. Another classical example is the production of putrescine. Putrescine is synthesized from agmatine, which is produced through the decarboxylation of arginine by the gut microbiota. In most cases, two metabolic pathways can be used to convert agmatine to putrescin: the agmatine ureohydrolase (SpeB) pathway and the N-carbamoylputrescine pathway. However, Kitada et al., found a novel putrescine synthesis pathway that requires the cooperation of two different bacterial species, neither of which express a complete agmatine biosynthetic pathway. Briefly, one species (such as *E. coli*) converts arginine to agmatine, and the other species (such as *Enterococcus faecalis*) yields putrescine from agmatine via agmatine deaminase [119]. More generally, cross-feeding of breakdown products in the gut environment is critical for various gut bacterial inhabitants.

Syntrophic cross-feeding for interspecies metabolic exchanges is very common in natural communities, and such exchanges offer significant advantages under poor nutrient conditions [112]. This cross-feeding can also balance cooperative interactions in the gut microbiota and play an important role in regulating intestinal nutrient absorption [120].

## Metabolites and the host immune system

**Fig. 3:**
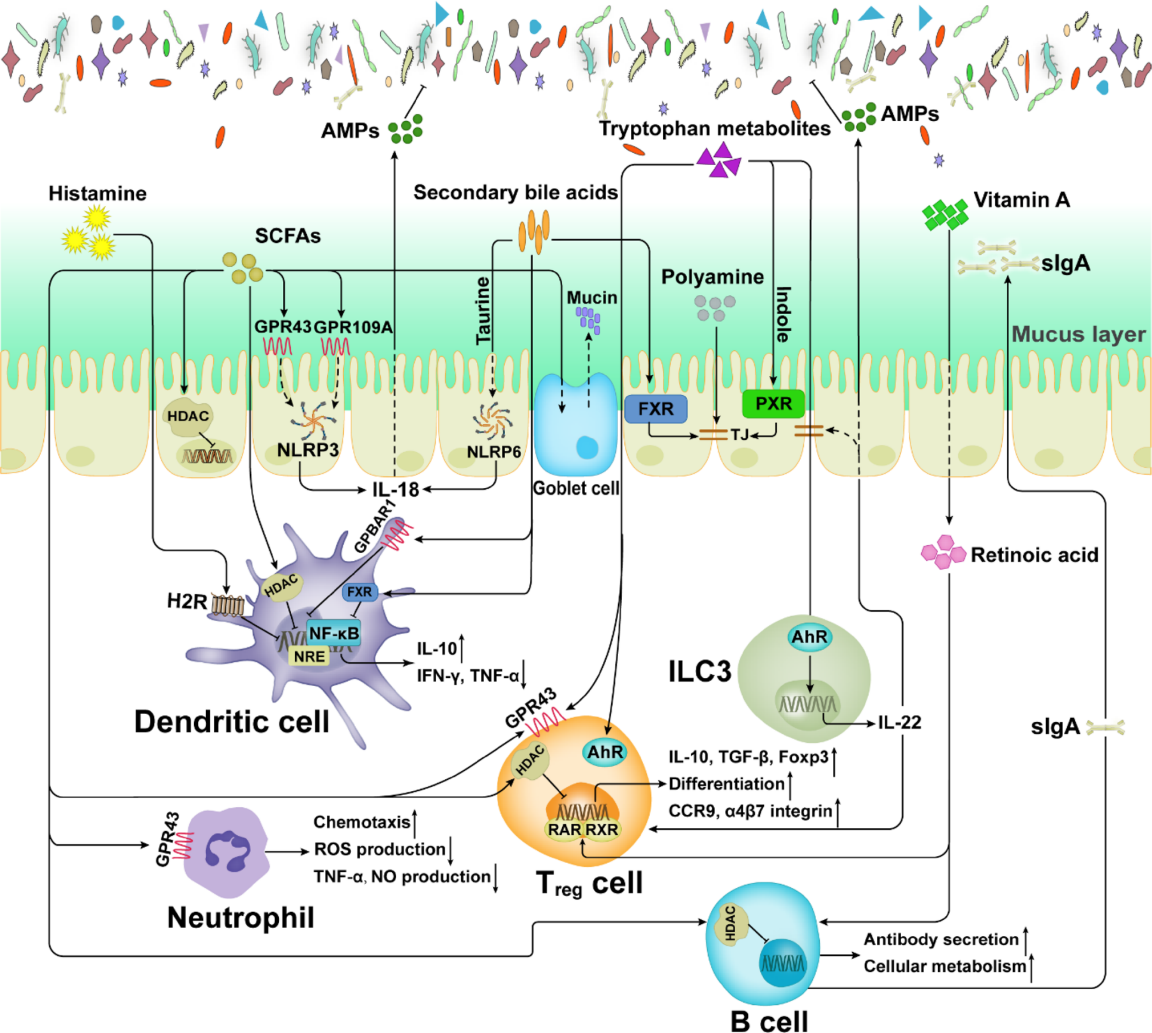
Effects of metabolites on immune cells. Metabolites derived from the microbiota or host participate in complicated host-microbiota interactions. *SCFAs* short chain fatty acids, *AMPs* antimicrobial peptides, *AhR* aryl hydrocarbon receptor, *FXR* farnesoid X receptor, *PXR* pregnane X receptor, *HADC* histone deacetylase, *TJ* tight junction, *ILC3* group 3 innate lymphoid cell

The intestinal microbiota helps to regulate many host physiological processes, such as nutritional homeostasis, energy expenditure, and immunity [121]. In recent years, several studies have revealed that the microbiota plays a critical role in host immune development and immune regulation. This intimate crosstalk may be driven by the secretion and signaling of small molecule metabolites and has profound impacts on host immunity and physiology [122]. As mentioned above, many microbiota-associated metabolites are bioactive, such as SCFAs, indole, and secondary bile acids, and these metabolites can react with corresponding sensing platforms in the host through signaling pathways [123] (Fig. 3).

Bioactive metabolites affect the maturation, activation, polarization, and effector functions of innate and adaptive immune cells, thereby modulating anti-inflammatory or pro-inflammatory responses (Table 2). In the next sections, we will provide an overview of the effects of microbiota-associated metabolites on intestinal immune cell subsets and their functions.

**Table 2:** Effects of metabolites on immune cells

Immune cell	Molecular mechanism(s)	Immunomodulatory metabolite	Effects on immune function	Key references
Interactions with innate immune cells				
Intestinal epithelial cells (IECs)	Inhibition of histone deacetylase. Activation of the NLRP3 inflammasome (butyrate only)	Short-chain fatty acids (acetate, propionate, butyrate)	Provides energy. Induces goblet cell differentiation and mucus expression. Strengthens tight junctions. Promotes production of IL-18	[121, 124–127]
	Activation of the NLRP6 inflammasome	Taurine	Enhances epithelial barrier function and production of IL-18	[128]
	Activation of FXR	Secondary bile acids	Promotes epithelial barrier function	[129, 130]
	Activation of PXR	Indole	Reinforces tight junctions	[131]
	Unclear. Inhibition of pro-inflammatory cytokines expression	Polyamines	Increases production of occludin, zonula occludens 1 and E-cadherin	[132]
Innate lymphoid cells (ILCs)	Activation of AhR	Indole and indole derivatives	Promotes expression of IL-22, increasing production of antimicrobial peptides and strengthening intestinal mucosa integrity. Protects against colitis	[133–136]
Dendritic cells (DCs)	Inhibition of histone deacetylase. Binding to the transporter Slc5a8 (propionate and butyrate only)	Short-chain fatty acids (acetate, propionate, butyrate)	Inhibits expression of pro-inflammatory cytokines (TNF-α, IL-12 and IFN-γ) and promote production of anti-inflammatory cytokines (IL-10). Inhibits differentiation from bone-marrow precursors and expression of co-stimulatory proteins	[84, 137–139]
	Activation of H2R	Histamine	Protects against colitis. Inhibits expression of pro-inflammatory cytokines and the MAPK pathway	[140]
	Activation of GPBAR1 and FXR	Secondary bile acids	Inhibits NF-κB-dependent transcription of proinflammatory genes	[141]
Neutrophils	Activation of GPR43. Inhibition of histone deacetylase	Short-chain fatty acids (acetate, propionate, butyrate)	Promotes chemotaxis. Suppresses activation of NF-κB and expression of NO. Regulates production of ROS	[142–145]
Interactions with adaptive immune cells				
T helper (Th) cells	Activation of AhR	Indole-3-lactic acid	Inhibits polarization of Th17 cells	[146]
	Activation of H2R or H1R	Histamine	Suppresses or promotes Th1 and Th2 polarization	[147]
Regulatory T (Treg) cells	Activation of GPR43 and GPR41. Inhibition of histone deacetylase	Short-chain fatty acids (acetate, propionate, butyrate)	Stimulates proliferation. Regulates the mTOR pathway for generation of IL-10	[148–150]
	Activation of RAR and RXR heterodimer	Retinoic acid	Activates the TGFβ–SMAD pathway. Increases expression of gut-homing markers	[151, 152]
	Increase expression of the antiapoptotic factor BCL-2	Folic acid	Promotes cell survival	[153, 154]
	Activation of GPR35, GPR109A and AhR	Tryptophan metabolites	Induces differentiation	[155, 156]
B cells	Inhibition of histone deacetylase	Short-chain fatty acids (acetate, propionate, butyrate)	Enhances oxidative phosphorylation, glycolysis and fatty acid synthesis. Promotes antibody production	[157, 158]
	Unclear	Retinoic acid	Facilitates IgA class-switch recombination and IgA production	[159]

### Interaction between metabolites and innate immunity

#### Intestinal epithelial cells (IECs)

The intestinal tract plays a key role in protecting the host from the environment and mediating nutrient absorption. The intestinal surface is composed of IECs, which form an important physical barrier that separates the lumen from the LP of intestinal and commensal bacteria [131]. Although IECs are not classical innate immune cells, they are a critical part of the mucosal immune system. Indeed, IECs are equipped with an extensive repertoire of innate immune receptors, which contribute to gut homeostasis through bacterial recognition [160].

SCFAs are the products of nondigestible carbohydrate fermentation by the intestinal microbiota. The host recognizes SCFAs by several G-protein-coupled-receptors (GPR41, GPR43 and GPR109A) [148, 161]. SCFAs serve as energy substrates for IECs, especially butyrate, which can modulate energy metabolism in IECs [124]. However, butyrate functions as not only as an energy source for colonocytes, but as also an inhibitor of intestinal stem cells. Butyrate suppresses proliferation by inhibiting histone deacetylase (HDAC) and increasing the promoter activity of cell-cycle negative regulators [125]. Therefore, the utilization of butyrate by enterocytes limits intestinal stem cell access to metabolites and protects these cells from anti-proliferative effects. SCFAs promote the transcription of mucin genes in epithelial goblet cells, and inoculation of several SCFA-producing bacteria in germ-free mice induces the differentiation of goblet cells and secretion of mucus [126, 162].

The inflammasome is a critical innate immunity sensing complex of IECs that regulates interactions between the host and microbiota by producing cytokine IL-18 and downstream antimicrobial peptides and secreting mucus [127, 163]. Butyrate influences the NLRP3 inflammasome by activating GPR43 or GPCR109A in IECs, facilitating the expression of the downstream inflammatory cytokine IL-18 and thus promoting epithelial repair and maintaining barrier function [121, 127]. Activation of the NLRP6 inflammasome is regulated by taurine, histamine, and spermine, whereas spermine and histamine can inhibit NLRP6 inflammasome signaling [128, 164].

Additionally, secondary bile acids (DCA and LCA) regulate epithelial cell integrity and microorganism composition by binding to the nuclear receptor farnesoid X receptor (FXR) expressed in IECs [129]. Mice with FXR deficiency exhibit epithelial barrier disruption and imbalanced gut homeostasis [130]. Indole, an amino acid microbial-mediated metabolite, promotes epithelial barrier function through the pregnane X receptor (PXR), which contributes to the reinforcement of tight junctions. By increasing the production of occludin, zonula occludens 1 (ZO1) and E-cadherin, polyamines are also responsible for enhancing the integrity of the IEC barrier [132, 165].

#### Innate lymphoid cells (ILCs)

Compared with adaptive lymphocytes, ILCs are relatively rare innate lymphocytes, but they are abundant on the barrier surface of mucosal-associated tissues [166, 167]. Recently, several studies reported that specific microbial metabolites can regulate ILCs, and most research has focused on ILC3 [168, 169], which produces the cytokine IL-22, a member of the IL-10 cytokine family. Lack of IL-22 is associated with different host pathologies (infections and metabolic disorders) [133, 170]. Furthermore, IL-22 promotes the expression of antimicrobial peptides (RegIIIγ and RegIIIβ) to limit colonization by commensal bacteria (such as SFBs), induces the fucosylation of surface proteins to promote the colonization of beneficial bacteria, and enhances the proliferation of goblet cells for mucin secretion [134–136].

AhR is a ligand-dependent transcription factor that plays a critical role in mucosal immunity. AhR ligands bind to the ligand-binding domain of the receptor, promoting conformational remodeling that exposes the nuclear localization sequence, and facilitates the transcription of target genes. The nuclear receptor RORγt is critical for the development of ILCs and RORγt^+^ ILCs are abundantly present in the intestinal LP [171–173]. In fact, without AhR, RORγt^+^ ILCs undergo higher rates of apoptosis and produce less IL-22. RORγt interacts with AhR to stimulate the binding of the latter at the IL-22 locus, promoting transcription [174]. Tryptophan catabolites can specifically regulate ILC3. As indicated above, tryptophan can be converted to active substances by both microorganisms and hosts, and most of these metabolites are AhR agonists (Fig. 1). Tryptophan acts as a metabolic substrate for the production of AhR ligands, such as indole-3-aldehyde and indole-3-lactic acid, by members of *Lactobacillus*, and these ligands help to resist colonization by *C. albicans* and SFBs[65]. For instance, the *Lactobacillus* population in the *Car*d9^−/−^ mouse intestine was shown to be low, which led to impairment of tryptophan metabolism and thus decreased AhR ligand production. Such a defect further impairs IL-22 production and increases the susceptibility of mice to dextran sodium sulfate (DSS)-induced colitis [91]. In addition, the ability to produce AhR agonists, especially those derived from the microbiota, in the feces of irritable bowel disease (IBD) patients is impaired. In general, the balance of ILC3 populations and IL-22 production is deeply dependent on the ability to produce AhR agonists; accordingly, these endogenous microbe-derived tryptophan metabolites are crucial for resistance to colonization and maintenance of gut immune homeostasis.

### Dendritic cells (DCs)

DCs are professional antigen-presenting cells that play a key role in bridging the innate and adaptive immune system. The immunomodulatory effects of DCs depend on producing cytokines and inducing the polarization and differentiation of naive CD4^+^ T lymphocytes [175]. The activation, survival, and maturation of DCs are processes that are influenced by mainly local factors within their microenvironment, such as microbial components, cytokines, and metabolites [176].

SCFAs act as HDAC inhibitors, and when exposed to DCs, SCFA-driven HDAC suppression is critical for inhibiting nuclear factor-κB (NF-κB) and tumor necrosis factor-α (TNF-α) [177, 178]. Propionate and butyrate inhibit bone-marrow precursor differentiation into DCs via their transporter Slc5a8 [137, 138], and exposure of DCs to butyrate is accompanied by decreased production of the inflammatory cytokines IL-12 and IFN-γ, and increased production of IL-10 and IL-23 [179]. Some evidence also indicates that butyrate may modulate the ability of DCs to present antigens and induce T cell differentiation. For instance, treatment of DCs with butyrate reduced the expression of co-stimulatory proteins, including CD40, CD80, CD86 and major histocompatibility complex class II molecules [139].

Histamine is a biogenic amine converted from the amino acid histidine via decarboxylase (hdc) (Table 1) [147]. Histamine regulates the responses of DCs to microbial ligands through H2R and then enhances the DC antigen-presenting capacity [140]. Administration of a histamine-producing *Lactobacillus rhamnosus* strain to H2R-knockout mice reduces the microbe’s immunoregulatory activities[84]. Additionally, *L.* *reuteri*-derived histamine suppresses the production of TNF-α and inhibits MEK/extracellular signal-regulated kinase mitogen-activated protein kinase (MAPK) signaling.

Secondary bile acids act as agonists of G-protein-coupled bile acid receptor 1 (GPBAR1) and nuclear receptor subfamily 1, group H, member 4 (NR1H4; also known as FXR), regulating the function of DCs [180, 181]. These receptors are linked to anti-inflammatory responses, including inhibition of NF-κB activity and NF-κB-dependent transcription [141, 182]. GPBAR1 signaling leads to the cAMP-PKA-regulated suppression of STAT1 and NF-κB, and NR1H4 signaling involves NR1H4-NCoR1-mediated repression of NF-κB-responsive elements (NRE) [183]. Activation of GPBAR1 leads to intracellular cAMP accumulation, further promoting the differentiation of CD14+ monocytes into CD209+ DCs [184].

#### Neutrophils

Neutrophils are the first effector cells that accumulate at an inflammation site, where they kill and digest bacteria [185]. In the case of mucosal infection or inflammation, neutrophils will pass through the LP and enter infected or inflamed sites. The migration of neutrophils depends on multiple factors, such as chemokines and integrins [186, 187]. After migrating to the designated location, activated neutrophils produce nitric oxide (NO), chemokines (e.g., IL-8) and pro-inflammatory factors (e.g., TNF-α and IL-1β), leading to maintenance of the inflammatory response as well as mucosal injury [188].

Several reports have shown that SCFAs are effective activators of neutrophils [148, 188, 189], as in vitro experiments have demonstrated that SCFAs induce neutrophil chemotaxis by binding to GPR43. In turn, GPR43-dependent signaling leads to the phosphorylation and activation of p38 MAPK [188], and p38 MAPK phosphorylation has been described as the major determinant of chemotaxis [142]. Compared with normal bone marrow-derived neutrophils (BMDNs), Gpr43^−/−^ BMDNs do not exhibit calcium influx and chemotaxis when treated with acetate [190]. Furthermore, Gpr43^−/−^ colitis mice treated with SCFAs (acetate or butyrate) showed reduced colonic neutrophil contents compared with those of wild-type colitis mice [191]. The inhibition of HDAC activity by SCFAs also suppress of NF-κB activation and NO production in neutrophils. Similar results were observed after exposure to HDAC inhibitors [143]. Nonetheless, SCFA specificity exists in reactive oxygen species (ROS) production, with acetate promoting production, butyrate inhibiting production, and propionate having no effect [144, 145]. The ROS produced by neutrophils help to protect against microbiota, modulate intracellular signaling activity, and regulate the inflammatory process [192, 193].

### Interactions between metabolites and adaptive immunity

The effects of commensal microbiota on the immune system include not only innate immunity but also adaptive immune responses. For example, germ-free mice have an immature adaptive immune system [28]. Although the molecular mechanism responsible for the effect of the microbiota on immune system development is still largely unknown, several metabolites have been shown to regulate adaptive immune cell function, especially those of CD4^+^ T and B lymphocytes.

### CD4^+^ T lymphocytes

As we previously discussed above, individual commensal species help to modulate polarization of the CD4^+^ T-cell compartment to defined CD4^+^ T cell subsets, including Th cells (Th1, Th2, Th17 cells) and Tregs. In generally, immune homeostasis and tolerance in the gut constitute the balance between pro-inflammatory effector Th cells and anti-inflammatory Tregs.

#### T helper (Th) cells

IFN-γ secretion by antigen presenting cells acts on original Th-cell signal transduction and transcription activating factor 1 (STAT-1). STAT-1 activates the specific transcription factor T-bet and prompts the differentiation of original Th cells into Th1 cells. Thl and Th2 cells are dynamically balanced under normal conditions, and are regulated and inhibited by cytokines secreted by each other. IFN-γ secreted by Th1 cells inhibits the polarization of Th2 cells, while IL-4 produced by Th2 cells inhibits Th1 proliferation. The imbalance of Th1/Th2 cells is associated with a variety of autoimmune diseases and inflammatory diseases, such as IBD [194]. The activation of different receptors in cell surface or intracellular could cause different activities. For example, the activation of H1R on lymphocytes promotes TH1 polarization, while the activation of H2R suppresses TH1 and TH2 polarization [147]. Histamine has important regulatory effects on T lymphocytes that are dependent on receptors.

Th17 cells are a CD4^+^ T-cell polarization in addition to Th1 and Th2, which are also pro-inflammatory effector Th cells. Th17 cells mainly function through secreting the cytokines, IL-17, IL-23 and IL-22, and pro-inflammatory mediators IL-17 and IL-23 can activate NF-κB and related inflammatory signaling pathway as well as block the expression of the anti-inflammatory cytokines IL-10 [195]. AhR expression is reduced in pathogenic Th17 cells, which leads to tissue inflammation. The AhR ligand indole-3-lactic acid was shown to inhibit the polarization of mouse Th17 cells in vitro [146]. In addition to the direct regulation of CD4^+^T cells function by microbial metabolites, the cytokines produced by IECs, DCs or macrophages induced by microbiota signals also play a significant role in CD4^+^ T-cell functionality [196, 197].

#### Regulatory T (Treg) cells

In the intestinal LP, many unique lymphocytes operate cooperatively to defend against infectious pathogens and maintain the epithelial mucosal barrier. However, an unrestrained inflammatory response to the ingestion of food and resident commensal microbiota by effector T cells or myeloid cells is harmful to intestinal health and immune homeostasis [198]. To maintain immune homeostasis, the immune system must tolerate antigens derived from food and gut microbiota, which partially depend on inducible Tregs. Tregs are characterized by the expression of CD4, CD25 and Foxp3 as well as the production of the anti-inflammatory cytokines TGF-β and IL-10. The development of intestinal Treg cells is affected by several metabolites derived from the microbiota and host.

As mentioned above, SCFAs have many effects on the innate immune system, and profound impact Treg biology. Several studies have demonstrated that SCFAs affect the number of Foxp3^+^/IL-10^+^ colonic Tregs and enhance the regulation of Tregs in the large intestine [149]. Further research has revealed that SCFAs stimulate the proliferation of Tregs by activating GPR43 or GPR41 [103] and the differentiation of naive CD4^+^ T cells into Tregs by inhibiting HDAC (e.g., HDAC6 and HDAC9) activation [149]. HDAC suppression in T cells enhances the acetylation of p70 S6 kinase and phosphorylation of rS6, and further regulates the mTOR pathway associated with the generation of IL-10 + T cells [150]. Interestingly, different SCFAs modulate Tregs through different mechanisms; acetate and propionate stimulate the expansion of preexisting colonic Treg cells (cTregs), whereas butyrate increases the de novo differentiation of naïve T cells toward Tregs [199]. In addition to directly regulating the responses of T cells, SCFAs modulate DC-T cell interactions, e.g., inhibiting the expression of the NF-κB component RelB through HDAC inhibition and inducing anti-inflammatory genes through GPR109A activation in DCs, thereby resulting in Tregs differentiation [164].

Retinoic acid (RA), a metabolite of dietary vitamin A catalyzed via aldehyde dehydrogenase (ALDH), plays a crucial role in mediating Treg expansion [200, 201]. In combination with TGFβ, RA induces the differentiation of peripherally derived Treg cells (pTregs) [202, 203]. In addition, by binding to RA receptor (RAR) and retinoid X receptor (RXR) heterodimers, RA activates TGFβ–SMAD signaling to promote Foxp3 transcription [151, 152]. RA also enhances the generation of RORγt^+^Foxp3^+^ pTregs in vitro, and inhibiting RA signaling prevents the development of these cells in vivo [204, 205]. RA increases the expression of gut-homing markers (e.g., CCR9 and α_4_β_7_ integrin) on pTregs, which helps naive T cells migrate to various tissue sites [202, 206, 207]. Other vitamins that regulate interactions between the gut microbiota and host mostly belong to the B and K groups, which the host cannot synthesize and must depend on commensal microorganisms for production [208, 209]. For instance, the water-soluble vitamin B9 (folic acid), which plays a critical role in maintaining Foxp3^+^ Treg cell homeostasis, can be synthesized by several bacteria (e.g., *Bifidobacterium* and *Lactobacillus*) [210–213]. FA promotes the survival of Foxp3^+^ Tregs by upregulating the expression of the antiapoptotic factor BCL-2. Mice fed an FA-deficient diet exhibit a reduced number of Foxp3^+^ Tregs in the intestinal LP and a higher susceptibility to colitis [153, 154].

As discussed above, most tryptophan metabolites are AhR ligands. However, some host-derived tryptophan metabolites (such as KA and niacin) can also act as GPR35 and GPR109A agonists to induce colonic Treg differentiation [155]. The *L. reuteri*-derived AhR agonist indole-3-lactic acid reprograms intraepithelial CD4^+^ T cells into CD4^+^ CD8αα^+^ T cells [156]. Polyamines have also been reported to be important for accelerating the maturation of LP CD4^+^ T cells to regulate adaptive immunity in rats [199, 214, 215]. In addition to its effects on the cytokine secretion of DCs, histamine also influences the function of Tregs depending on the receptors they express [216]; for example, the activation of H1R expressed on Tregs leads to inhibition of the suppressive functions of these cells, as related to the decreased expression of CD25 and Foxp3[217].

#### B cells

Immunoglobulin secretion plays a critical role in immune regulation and protection against microorganisms in the intestine. Secretory immunoglobulin A (SIgA) populations comprise the largest class of immunoglobulins in the intestinal mucosal [33]. SIgA is secreted by plasma cells (differentiated B cells) in the LP and then passes through the intestinal epithelium into the lumen, where it targets microbial antigens and prevents bacterial translocation and infection [16].

Dietary fiber intake is positively correlated with intestinal IgA levels [218, 219]. One study revealed that SCFAs are able to regulate B-cell gene expression via HDAC inhibition to promote antibody secretion, similar to the effect of SCFAs on other cells [157]. Further research has shown that SCFAs modulate metabolic sensors to enhance oxidative phosphorylation, glycolysis and fatty acid synthesis in B cells [158]. These functions increase mitochondrial energy production and the levels of building blocks to promote B-cell activation, differentiation, and antibody production [220]. RA also modulates the activity of B cells, directly affecting their ability to facilitate IgA class-switch recombination and IgA production [159]. Nonetheless, as the role of microbial metabolites in the regulation of antibody production in B cells remains largely unclear, more research on the regulation of commensally derived metabolites of host antibody responses is needed.

### Microbiota-gut-brain axis: maintenance of immune homeostasis

The gut and brain are intimately connected by the gut-brain axis, which is a complex bidirectional adjustment system that includes the central and enteric nervous systems [221, 222]. Signals from the brain, and vice versa, influence the sensory and secretory modalities of the gastrointestinal tract, regulate the inflammatory process and influence the gut microbiota structure [223]. In turn, messages secreted from the gastrointestinal tract can influence brain function. A dysbiotic microbiota (associated with a high-sugar diet) reportedly alters vagal gut-brain communication [224]. The immune system plays a vital role in gut-brain axis communication, as immune mediators are important messengers in this complex dialog, which may explain the functional impairments in both the brain and gut in some diseases (e.g., IBD and psychological morbidity) [225].

As previously described, the bioactive small molecule metabolites originating from the gut microbiota can maintain intestinal immune homeostasis dependent on lymphocytes. In recent years, increasing evidence supports that these microbial products can enter the circulatory system and prominently affect the inflammatory responses of resident cells in the central nervous system (CNS). While SCFAs have immunomodulatory effects on the gut, they can also permeate the mucosa into the LP and enter systemic circulation [226]. Acetate and propionate, but not butyrate, can be detected in the peripheral circulation, indicating that distinct SCFAs have different functions in the immune system [227, 228]. SCFAs can also directly signal to the CNS depending on stimulation of the vagus nerve or exert indirect effects through immune-neuroendocrine processes [229]. Systemic SCFAs are capable of increasing the integrity of the blood–brain barrier by upregulating of the tight junction protein occludin [230]. Prebiotics such as inulin increased the production of acetate by crossing the blood–brain barrier in rats and affecting physiological function [231]. Tryptophan metabolites produced by the microbiota suppress NF-κB signaling activation, TGFα and VEGF-B production in microglia via activating AhR [232]. Lower VEGF-B expression limits the transcriptional programming of inflammatory astrocytes and thus limits their ability to produce damaging metabolites, such as NO, which recruit peripheral immune cells, and activate T and B cells in the CNS, thus amplifying local inflammation [196]. Indole, indoxyl-3-sulfate, indole-3-propionic acid and indole-3-aldehyde cross the blood–brain barrier and suppress inflammation in combination with IFN-I in astrocytes [13].

## Concluding remarks

Scientific research has revealed the complexity and breadth of interactions between commensal microorganisms and hosts. Metabolites derived by the microbiota serve as chemical messengers that mediate crosstalk between the microbes and host and play beneficial and deleterious roles in host health. In fact, the intestinal microbiota appears to affect many human disorders (e.g., IBD, cancer and allergies) [233].

Identifying the molecular mechanisms by which the gut microbiota or microbial metabolites modulate host health is important for exploring new approaches for disease research. However, research on the regulation of host-microbe interactions by metabolites has focused on only certain aspects, such as intestinal immunity and microbial composition, and the in-depth molecular mechanism remains unclear. Small molecule metabolites, such as indole, also act as signaling molecules for interbacterial communication and quorum sensing, thereby driving changes in the function and composition of the microbiota to modulate intestinal homeostasis. Overall, interactions between the host and the microbiota are complex and variable. The following questions need to be answered: (1) Can metabolites originate from the host, the microbiota or their common regulation, and how can these sources be distinguished? (2) Can some factors (e.g., diet, age, environment, and mental health) affect gut microbiota composition, and in turn affect the production of microbial metabolites? (3) What are the structures and metabolic synthesis mechanisms of these unknown biologically active microbial metabolites? (4) What interactions exist between multiple metabolites from different microbiota?

Fortunately, new approaches (e.g., multiomics approaches) have begun to unravel these complex host-microbiota interactions and further our knowledge of the mechanism involved. To complement traditional metabolomics, metagenomics helps to elucidate the mechanisms underlying metabolites biosynthesis through a bioinformatics perspective and discover more new biologically active microbial metabolites [233].

Using small molecules from microbiota as drugs to reduce inflammation and disease severity is promising but basic research and clinical data with complex traits are still required. Additionally, because of microbiota and genetic differences between individuals, personalized precision medicine should be considered [234].

Thus, a deeper understanding of the effects of metabolites on host homeostasis and disease may provide an opportunity for the administration of small molecule metabolites as prophylactic or therapeutic treatments in microbiota-related diseases.
